# A novel pathogenic *MLH1 *missense mutation, c.112A > C, p.Asn38His, in six families with Lynch syndrome

**DOI:** 10.1186/1897-4287-8-7

**Published:** 2010-08-12

**Authors:** Els van Riel, Margreet GEM Ausems, Frans BL Hogervorst, Irma Kluijt, Marielle E van Gijn, Jeanne van Echtelt, Karen Scheidel-Jacobse, Eric FAM Hennekam, Rein P Stulp, Yvonne J Vos, G Johan A Offerhaus, Fred H Menko, Johan JP Gille

**Affiliations:** 1 Department of Medical Genetics, University Medical Centre Utrecht, Lundlaan 6, Utrecht, The Netherlands; 2 Family Cancer Clinic and Department of Pathology, The Netherlands Cancer Institute, Plesmanlaan 121, Amsterdam, The Netherlands; 3 Department of Pathology, St. Antonius Hospital, Koekoekslaan 1, Nieuwegein, The Netherlands; 4 Department of Genetics, University Medical Centre Groningen, University of Groningen, Hanzeplein 1, Groningen, The Netherlands; 5 Department of Pathology, University Medical Centre Utrecht, Heidelberglaan 100, Utrecht, The Netherlands; 6 Department of Clinical Genetics, VU University Medical Centre, De Boelelaan 1117, Amsterdam, The Netherlands

## Abstract

**Background:**

An unclassified variant (UV) in exon 1 of the *MLH1 *gene, c.112A > C, p.Asn38His, was found in six families who meet diagnostic criteria for Lynch syndrome. The pathogenicity of this variant was unknown. We aim to elucidate the pathogenicity of this *MLH1 *variant in order to counsel these families adequately and to enable predictive testing in healthy at-risk relatives.

**Methods:**

We studied clinical data, microsatellite instability and immunohistochemical staining of MMR proteins, and performed genealogy, haplotype analysis and DNA testing of control samples.

**Results:**

The UV showed co-segregation with the disease in all families. All investigated tumors showed a microsatellite instable pattern. Immunohistochemical data were variable among tested tumors. Three families had a common ancestor and all families originated from the same geographical area in The Netherlands. Haplotype analysis showed a common haplotype in all six families.

**Conclusions:**

We conclude that the *MLH1 *variant is a pathogenic mutation and genealogy and haplotype analysis results strongly suggest that it is a Dutch founder mutation. Our findings imply that predictive testing can be offered to healthy family members. The immunohistochemical data of MMR protein expression show that interpreting these results in case of a missense mutation should be done with caution.

## Background

About 3% of all colorectal cancers is due to Lynch syndrome, an autosomal dominant condition caused by germline mutations in one of the DNA mismatch repair (MMR) genes, *MLH1*, *MSH2, MSH6 *and *PMS2 *[1]. Carriers of a mutation in one of these MMR genes have a high risk of developing colorectal cancer, endometrial cancer and also an increased risk of specific other malignancies including ovarian, upper urinary tract, gastric, small intestinal and biliary tract cancer and adenoma or carcinoma of the sebaceous gland [1].

In families who meet the Amsterdam and/or the revised Bethesda criteria [2], tumor examination is indicated including an assay for microsatellite instability (MSI) and immunohistochemistry (IHC) for mismatch repair (MMR) protein expression [2]. When a tumor shows MSI, with or without alterations in immunohistochemical staining of these proteins, mutation analysis of the *MLH1*, *MSH2*, *MSH6 *and *PMS2 *genes is offered.

Missense mutations comprise about 20% of all pathogenic mutations associated with Lynch syndrome [3,4]. For most missense mutations, convincing evidence for pathogenicity is lacking, and these are called unclassified variants (UVs) or variants of uncertain clinical significance [3,5,6]. To gain more insight in the nature of such an UV, it is useful to study clinical, morphological and molecular features of affected patients and their families.

In this study, we describe six Dutch families with Lynch syndrome and a previously described UV in the *MLH1 *gene [7-10]. We have combined data from literature with several parameters studied in these families like co-segregation of the MSI/IHC results and the UV with disease, germline mutation analysis of MMR genes, haplotype analysis, geneaology, and germline mutation testing of healthy controls to gain more insight into the clinical significance of this UV.

## Methods

### Patients and families

**Figure 1:**
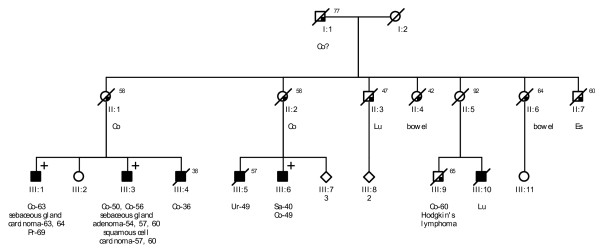
**Pedigree from Family 1 (VUmc C198)**. Co = colon cancer. Lu = lung cancer. Es = oesophageal cancer. Pr = prostate cancer. Ur = ureteral cell cancer. Sa = sarcoma.

**Figure 2:**
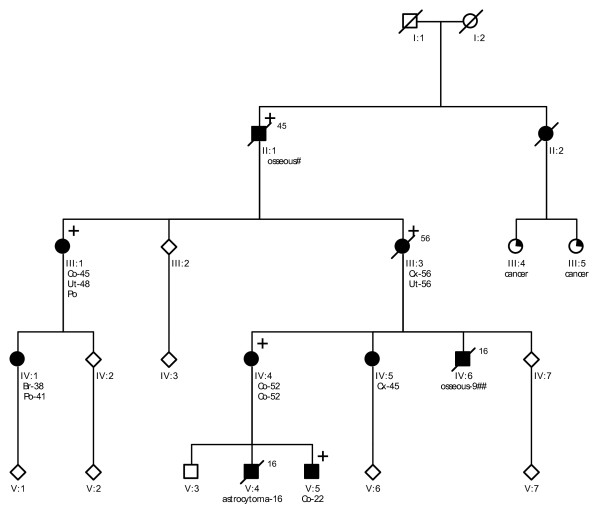
**Pedigree from Family 2 (UMCU1)**. Co = colon cancer. Ut = endometrial cancer. Po = colonic polyp. Br = breast cancer. Cx = cervical cancer. # 'cancer of bones' according to family; no review of pathology. ## 'cancer of bones' according to family; consistent with neuroblastoma according to pathology report.

**Figure 3:**
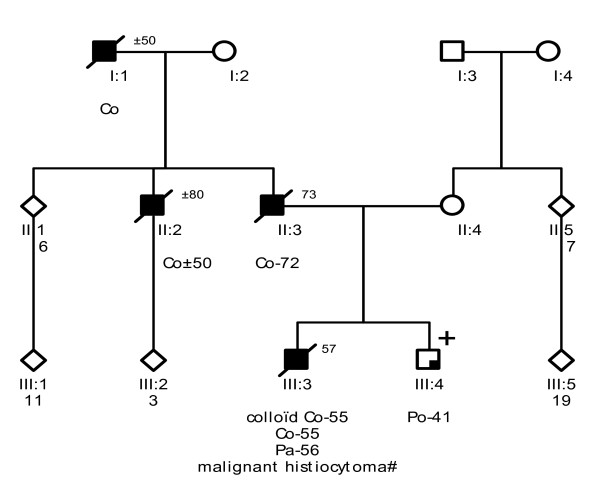
**Pedigree from Family 3 (UMCU2)**. Co = colon cancer. Pa = pancreatic cancer. Po = colonic polyp. # malignant histiocytoma as stated in a letter from 1974; no specification was given, pathology could not be reviewed.

**Figure 4:**
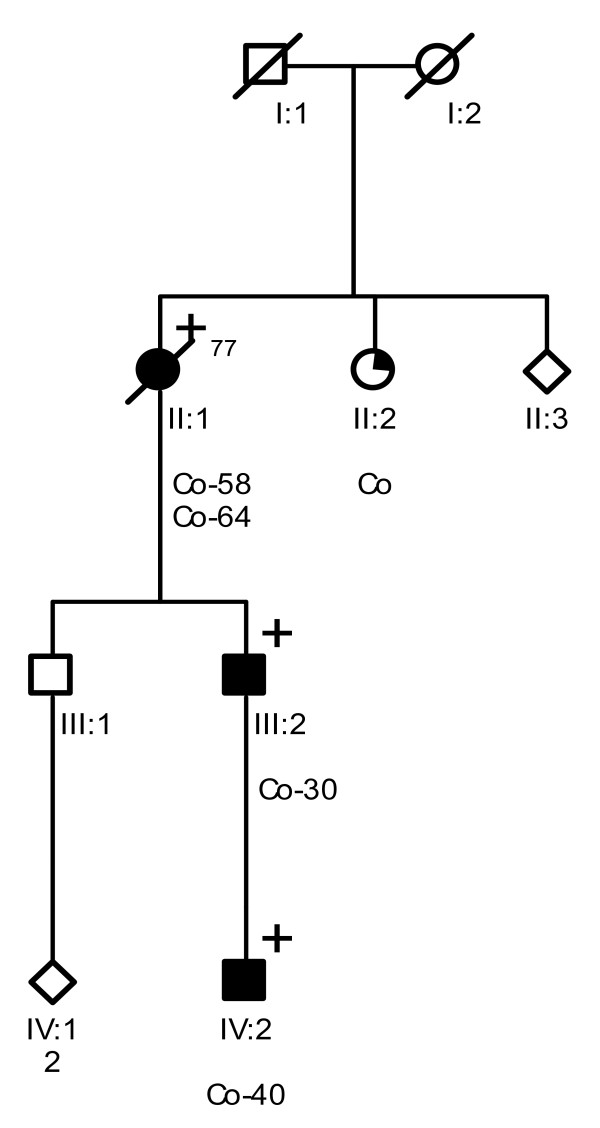
**Pedigree from Family 4 (NKI F1390)**. Co = colon cancer.

**Figure 5:**
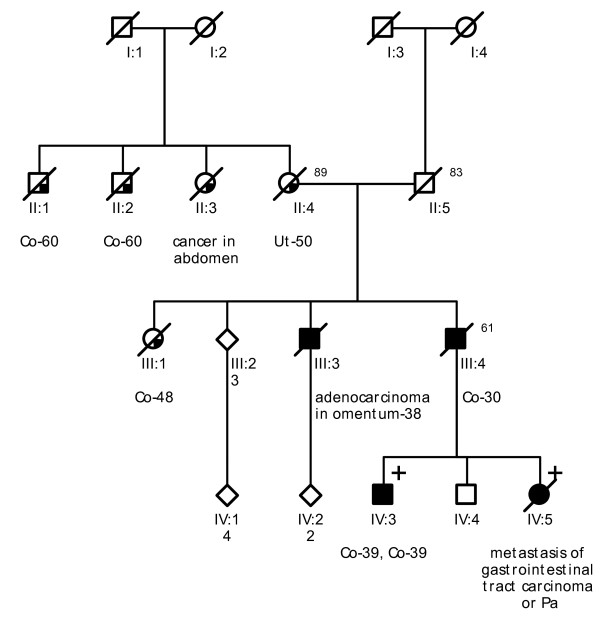
**Pedigree from Family 5 (UMCG1)**. Co = colon cancer. Ut = endometrial cancer. Pa = pancreatic cancer.

**Figure 6:**
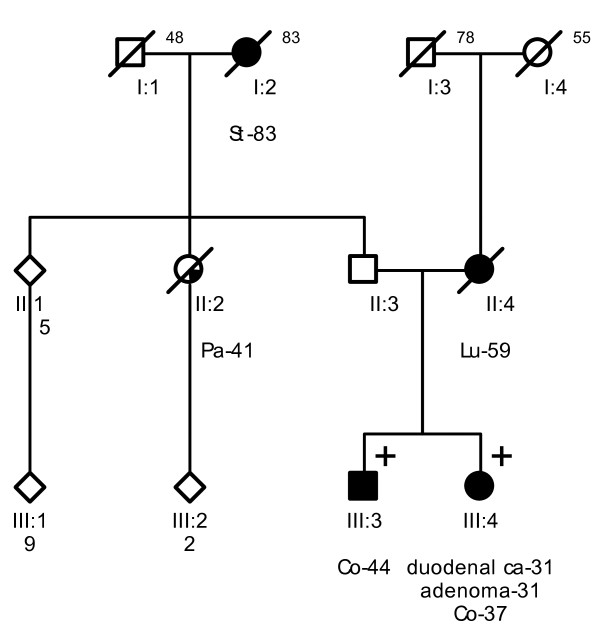
**Pedigree from Family 6 (UMCG2)**. St = gastric cancer. Pa = pancreatic cancer. Lu = lung cancer. Co = colon cancer. ca = carcinoma.

Probands of these six families were referred to a Family Cancer Clinic for genetic counselling because of a personal and/or family history of cancer. A detailed pedigree analysis was performed and, if possible, medical data of affected relatives were verified (Figure 1, Figure 2, Figure 3, Figure 4, Figure 5, Figure 6). For all affected relatives clinical data were recorded including sex, type of cancer, age at diagnosis or at death.

### Microsatellite instability analysis and immunohistochemistry for MMR proteins

For MSI analysis, genomic DNA was isolated from formalin-fixed and paraffin-embedded tumor and normal tissue using standard procedures [11-13]. At least five markers from the Bethesda panel with some additional markers were used to assess MSI. According to international guidelines, a tumor was considered having a MSI-high phenotype when at least two out of five markers were instable [12].

IHC was performed on formalin-fixed, paraffin-embedded tumor and normal tissues. Slides were stained following routine diagnostic procedures [13] using appropriate antibodies against *MLH1*, *MSH2*, *MSH6 *and, when available, *PMS2 *proteins.

The cancer specimens were acquired from different hospitals where the patients were treated. These pathology departments used a variety of protocols for formalin-fixation and paraffin-embedding. MSI and IHC were conducted on 18 different specimens of 13 patients, including 13 colorectal tumors (two of which only IHC and no MSI), 1 sebaceous gland carcinoma, 1 sebaceous gland adenoma (only MSI, no IHC), 1 endometrial carcinoma, 1 duodenal carcinoma and 1 metastasis presumably of a primary tumor of the gastrointestinal tract or pancreas.

### Mutation analysis

When a tumor was classified as MSI high, with or without alterations in immunohistochemical staining of one or more of the MMR proteins, germline mutation analysis was offered. DNA was isolated from peripheral blood lymphocytes of affected patients.

For some patients, a complete germline mutation analysis for not only *MLH1 *[GenBank: NM_000249] but also *MSH2 *[GenBank: NM_000251] and *MSH6 *[GenBank: NM_000179], and in some cases *PMS2 *[GenBank: NM_000535], was performed (see Additional file 1 - Table S1: Microsatellite instability- and immunohistochemistry results in patients with *MLH1 *missense mutation).

### Genealogy

Extended pedigrees of 6 generations were studied to investigate possible common ancestry of the six families.

### Shared haplotype analysis

Haplotype analysis was performed of all probands and affected relatives if available to investigate a possible common origin of the UV. The haplotype consists of 6 VNTR markers, with 3 markers flanking each side of the gene.

### Control population

Germline mutation analysis for this UV was performed by DNA sequencing in 94 healthy, anonymous Dutch controls.

## Results

### Clinical phenotype of families

All six families fulfilled the revised Bethesda criteria, while Families 1, 2 and 4 also fulfilled the Amsterdam II criteria. Figure 1, Figure 2, Figure 3, Figure 4, Figure 5 and Figure 6 show phenotypic details of these families.

Family 1 has features of Muir-Torre syndrome, a phenotypic variant of Lynch syndrome [14].

### MSI and IHC

All tested colorectal and other carcinomas showed a MSI-high phenotype. Of 17 tumors that were examined immunohistochemically for MMR protein expression, 6 stained negative for one or more MMR proteins. Only one of these was negative for *MLH1*. MSI and IHC results are summarized in Table S1 (see Additional file 1 - Table S1: Microsatellite instability- and immunohistochemistry results in patients with *MLH1 *missense mutation).

### Germline mutation analysis

In 13 affected patients an UV in exon 1 of the *MLH1 *gene, c.112A > C, p.Asn38His, was found. All tested affected patients carried the UV, indicating complete co-segregation of the UV with the disease. In some patients additional germline mutation analysis of the *MSH2*, *MSH6 *and/or the *PMS2 *gene was performed (see Additional file 1 - Table S1: Microsatellite instability- and immunohistochemistry results in patients with *MLH1 *missense mutation) and no mutations were found in these genes.

### Genealogy

**Figure 7:**
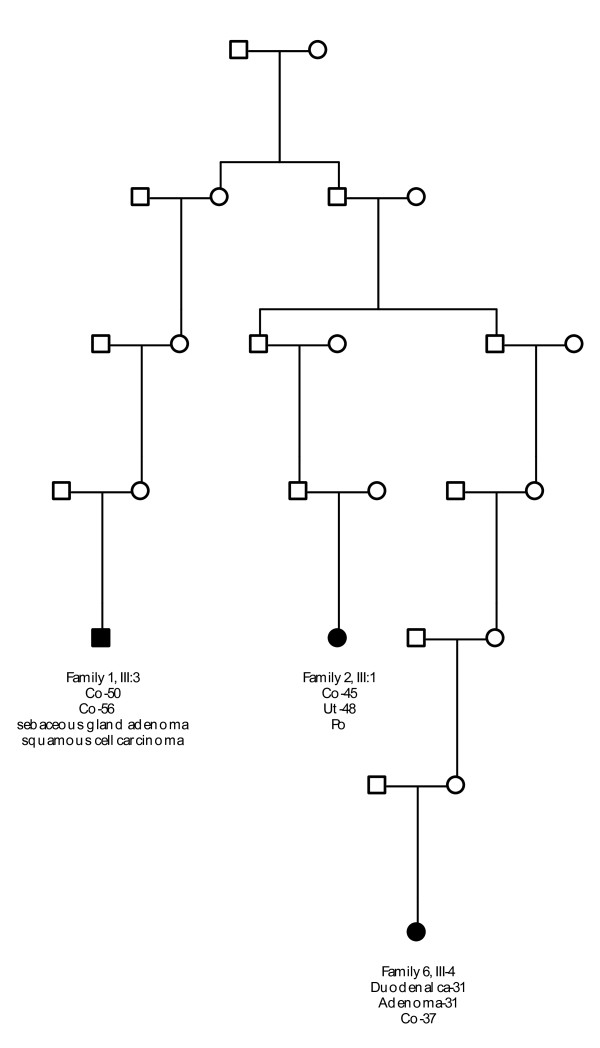
**Genealogy result**. Co = colon cancer. Ut = endometrial cancer. Po = colonic polyp. ca = carcinoma.

All ancestors of the six families originated from the same region (Mid-East Netherlands) and certain family names were frequently found in the different pedigrees. Three families (Families 1, 2 and 6) were shown to have a common ancestor, who linked the independently referred individuals of these families together into a seven-generation family (Figure 7).

### Haplotype analysis

All tested patients (n = 12) from six families were shown to share a common allele (see Additional file 2 - Table S2: Haplotype analysis results).

### Controls

The *MLH1 *variant c.112A > C was not detected in DNA of 94 healthy anonymous controls.

## Discussion

In this study, we show that the *MLH1 *alteration, c.112A > C (p.Asn38His), most likely represents a pathogenic missense mutation causing Lynch syndrome.

Criteria for pathogenicity of missense mutations include difference in the chemical properties of the amino acid (Grantham score), evolutionary conservation of the amino acid, absence in the normal population, co-segregation with the disease and association between MSI and/or absence of immunohistochemical staining for the MMR protein in the tumor [4]. From literature and available databases, it is known that this mutation lies in a functional domain of the *MLH1 *gene and the Asn-38 codon has a full evolutionary conservation in the *MLH1 *protein [3,8,15]. Furthermore, another missense mutation in this codon, p.Asn38Asp, c.112A > G, is proven to be pathogenic and affects the activity of the *MLH1 *protein: the protein cannot bind Mg++, does not have ATPase activity and cannot correct for a *MLH1 *deficiency [15]. Lack of mismatch repair activity has also been described recently for this p.Asn38Asp mutation [10]. The variant described in our study induces a greater shift in Grantham score than the variant described by Takahashi [15]. Also, according to SIFT and MAPP-MMR scores this UV should be considered pathogenic [9,10].

The results of our study show a complete co-segregation between the cancer cases (colorectal, endometrial and other Lynch syndrome related cancers) and the mutation in six families. All tumors show an MSI high phenotype, mostly without alterations in immunohistochemical protein expression. Pathogenic mutations are known to induce MSI and absence of immunohistochemical staining of one or more MMR proteins. In general, immunohistochemical staining is considered to be more sensitive in detecting MMR gene mutations than MSI analysis [16-18]. However, missense mutations can have subtle effects on the protein products of the MMR genes, which can result in loss of function while maintaining intact protein that is able to bind antibodies [19]. Therefore, immunochemistry can show normal staining of the proteins, as seen in the majority of the tumors in this study. In accordance to our findings, other studies have also described tumors showing an MSI high phenotype but normal expression of the MMR proteins [7,10]. We also observed loss of immunostaining of *PMS2 *in some tumors. Loss of *PMS2 *protein expression is often seen in tumors from *MLH1 *mutation carriers, since *PMS2 *and *MLH1 *form heterodimeric complexes in mismatch repair [19]. Surprisingly, we also observed loss of *MSH2 *and/or *MSH6 *protein expression. An artefact in the methods used in this study is unlikely since staining in normal tissue of the same patients was normal and others also have reported loss of *MSH2 *and *MSH6 *protein expression in a tumor of a *MLH1 *missense mutation carrier [19] and control tissue showed normal staining. Immunostaining can be dependent on a second hit which can differ for each tumor [20].

Taken together, in the case of missense mutations, which might lead to presence of abnormal protein products that still have binding capacity for antibodies, MSI might be more sensitive for detecting patients with MMR gene mutations than immunohistochemical staining [21,22].

All tested family members share a common allele and their ancestors originate from the same region in The Netherlands. Genealogical studies showed that three families have a common ancestor. The variant in our families is reported earlier by Van Puijenbroek [7], who studied genome-wide copy neutral loss of heterozygosity (LOH) in a cohort of Dutch familial and sporadic colorectal carcinomas. To our knowledge, this mutation has not been reported in families outside The Netherlands [3,10,23,24]. Together with the genealogy and haplotype analysis, this makes the mutation likely to be a Dutch founder mutation.

In the described families, a Lynch syndrome phenotype is clearly present. In addition to colorectal and endometrial cancer, other cancers were also present in mutation carriers: skin cancers (sebaceous gland carcinoma and squamous cell carcinoma), sarcoma, malignant histiocytoma, ureteral cell cancer, astrocytoma and bone tumors not otherwise specified. Most of these cancers fit within the tumor spectrum of Lynch syndrome. However, sarcoma and histiocytoma are not common tumor types seen in Lynch syndrome. There are some studies that suggest these tumors can be part of the tumor spectrum and a causal relationship with *MSH2 *has been shown [25-27]. In our study, we confirmed that the patient with the sarcoma is a carrier of the *MLH1 *mutation. We were not able to confirm that the patient affected with histiocytoma has been a carrier of the *MLH1 *gene mutation. In Family 1 (VUmc C198), the family history is consistent with Muir-Torre syndrome [14]. Sebaceous gland carcinomas are part of the Lynch syndrome tumor spectrum [28,29]. Our patients affected with sebaceous gland carcinoma and sebaceous gland adenoma were proven to be carriers of the *MLH1 *gene mutation and tumor specimens showed a MSI-high phenotype, which confirms the diagnosis Lynch/Muir-Torre syndrome.

## Conclusions

The results of our study show compelling evidence that the described missense mutation c.112A > C, p.Asn38His, affecting a strongly conserved position in the ATP-ase domain of the *MLH1 *gene, is indeed a pathogenic germline alteration which causes Lynch syndrome. Therefore, predictive testing can be offered to non-affected family members. In addition, the results of IHC are not unambiguous and apparently not reliable in diagnosing Lynch syndrome in at least a part of the families with missense mutations in MMR genes.

## Competing interests

The authors declare that they have no competing interests.

## Authors' contributions

EVR counseled part of the families and acted as first author on this manuscript. MGEMA has been involved in drafting the manuscript and revising it critically for important intellectual content. FBLH carried out part of the MSI/IHC analysis and initiated the first contact between different family cancer clinics to elucidate the nature of this UV. IK counseled part of the families and initiated the first contact between different family cancer clinics to elucidate the nature of this UV. MEVG carried out part of the germline mutation analysis and supervised part of the MSI/IHC analysis. JVE counseled part of these families and initiated revision of tumor material in some of the patients. KSJ carried out part of the MSI/IHC analysis. EFAMH carried out genealogic research on these families. RPS counseled part of the families and made it possible to include two families in this study. YJV carried out part of the germline mutation analysis and supervised part of the MSI/IHC analysis. GJAO carried out part of the MSI/IHC analysis and revised tumor material from some patients. FHM counselled part of the families, made a substantial contribution to conception and design of this study, and has been involved in revising the manuscript critically for important intellectual content. JJPG carried out part of the germline mutation analysis, haplotype analysis and germline mutation analysis of the Dutch controls and has been involved in drafting the manuscript and revising it critically for important intellectual content. All authors read, revised and approved the final manuscript.

## Supplementary Material

Additional file 1 **Table S1**. Microsatellite instability- and immunohistochemistry results in patients with ***MLH1 ***missense mutation and affected family members. Click here for file

Additional file 2 **Table S2**. Haplotype analysis results. Click here for file
