# Supplementary material for: A novel pathogenic MLH1 missense mutation, c.112A > C, p.Asn38His, in six families with Lynch syndrome
Source: Hered Cancer Clin Pract. 2010 Aug 12;8(1):7. doi: 10.1186/1897-4287-8-7 (PMC2927519; doi:10.1186/1897-4287-8-7)
Supplement: Additional file 1 — Table S1. Microsatellite instability- and immunohistochemistry results in patients with MLH1 missense mutation and affected family members. [file 1897-4287-8-7-S1.DOC]

Table 1

|  | **material** | **MSI** | **IHC** | | | | **Germline mutation analysis** |
| --- | --- | --- | --- | --- | --- | --- | --- |
|  |  |  | ***MLH1*** | ***MSH2*** | ***MSH6*** | ***PMS2*** |  |
| Family 1, III:1 | colon carcinoma  sebaceous gland carcinoma | np  high | +  + | +  + | +  + | +  + | *MLH1*^1^: c.112A>C (p.Asn38His)  *MSH2*: normal  *MSH6*: normal |
| Family 1, III:3 | colon carcinoma  colon carcinoma  sebaceous gland adenoma | np  high  high | +  +  np | +  +  np | -  +  np | np  +  np | *MLH1*^1^: c.112A>C (p.Asn38His)  *MSH6*: normal |
| Family 1, III:4 | colon carcinoma | high | + | + | + | np | np |
| Family 1, III:6 | sarcoma  colon carcinoma | np  np | np  np | np  np | np  np | np  np | *MLH1*^2^: c.112A>C (p.Asn38His) |
| Family 2, III:1 | colon carcinoma  endometrial carcinoma | high  high | +  + | +  + | -  + | -  + | *MLH1*^1^: c.112A>C (p.Asn38His)  *MSH2*: normal  *MSH6*: normal  *PMS2*: normal |
| Family 2, IV:4 | colon carcinoma | high | + | + | + | + | *MLH1*^1^: c.112A>C (p.Asn38His) |
| Family 2, V:5 | colon carcinoma | high | + | + | + | + | *MLH1*^2^: c.112A>C (p.Asn38His) |
| Family 2, III:3 | cervical carcinoma  endometrial carcinoma | np  np | np  np | np  np | np  np | np  np | Obligate carrier based on pedigree |
| Family 2, II:1 | osseous^#^ | np | np | np | np | np | Obligate carrier (based on genealogy results) |
| Family 3, III:3 | colon carcinoma (colloid) | high | + | + | + | - | np |
| Family 3, III:4 | tubular colonic polyp | np | np | np | np | np | *MLH1*^1^: c.112A>C (p.Asn38His)  *MSH2*: normal  *MSH6*: normal  *PMS2*: normal |
| Family 4, IV:2 | colon carcinoma | high | + | + | + | np | *MLH1*^1^: c.112A>C (p.Asn38His)  *MSH2*: normal |
| Family 4, III:2 | colon carcinoma | high* | + | - | - | np | *MLH1*^2^: c.112A>C (p.Asn38His) |
| Family 4, II:1 | colon carcinoma | high | - | - | - | + | *MLH1*^2^: c.112A>C (p.Asn38His) |
| Family 5, IV:3 | colon carcinoma | np | np | np | np | np | *MLH1*^2^: c.112A>C (p.Asn38His) |
| Family 5, IV:5 | metastasis of gastrointestinal tract carcinoma or pancreas carcinoma | high | + | + | + | + | *MLH1*^1^: c.112A>C (p.Asn38His)  *MSH2*: normal  *MSH6*: normal |
| Family 6, III:3 | colon carcinoma | high | + | + | + | - | *MLH1*^2^: c.112A>C (p.Asn38His) |
| Family 6, III:4 | duodenal carcinoma  colon carcinoma | high  high | +  + | +  + | +  + | +  + | *MLH1*^1^: c.112A>C (p.Asn38His) |

* Possible MSI-high, no normal tissue available

+: normal presence of immunostaining; -: absence of immunostaining; np: not performed

^1^: entire *MLH1* gene is sequenced, no additional sequence variants were found

^2^: directly tested for c.112A>C, p.Asn38His, in the *MLH1* gene

^#^ ‘cancer of bones’ according to family; no review of pathology
