# Supplementary material for: A novel pathogenic MLH1 missense mutation, c.112A > C, p.Asn38His, in six families with Lynch syndrome
Source: Hered Cancer Clin Pract. 2010 Aug 12;8(1):7. doi: 10.1186/1897-4287-8-7 (PMC2927519; doi:10.1186/1897-4287-8-7)
Supplement: Additional file 2 — Table S2. Haplotype analysis results. [file 1897-4287-8-7-S2.DOC]

*Table 2^1^*

| marker | Family 1 | | | Family 2 | |  | Family 3 | Family 4 | Family 5 | | Family 6 | |
| --- | --- | --- | --- | --- | --- | --- | --- | --- | --- | --- | --- | --- |
|  | III:1 | III:3 | III:6 | III:1 | IV:4 | V:5 | III:4 | IV:2 | IV:3 | IV:5 | III:3 | III:4 |
| D3S1298 | **197** - 213 | **197** - 213 | **197** - 197 | **197** - 201 | **197** - 197 | **197** - 211 | **197** - 207 | **197** - 199 | **197** - 195 | **197** - 195 | **197** - 199 | **197** - 207 |
| D3S3512 | **126** - 128 | **126** - 128 | **126** - 128 | **126** - 134 | **126** - 126 | **126** - 138 | **126** - 130 | **126** - 134 | **126** - 134 | **126** - 134 | **126** - 128 | **126** - 132 |
| D3S1561 | **226** - 226 | **226** - 226 | **226** - 226 | **226** - 226 | **226** - 226 | **226** - 226 | **226** - 226 | **226** - 226 | **226** - 248 | **226** - 248 | **226** - 226 | **226** - 226 |
| *MLH1* | **N38H** - wt | **N38H** - wt | **N38H** - wt | **N38H** - wt | **N38H** - wt | **N38H** - wt | **N38H** - wt | **N38H** - wt | **N38H** - wt | **N38H** - wt | **N38H** - wt | **N38H** - wt |
| D3S1611 | **260** - 260 | **260** - 260 | **260** - 260 | **260** - 260 | **260** - 264 | **260** - 260 | **260** - 264 | **260 - 254** | **260** - 264 | **260** - 264 | **260** -260 | **260** - 264 |
| D3S3623 | **223** - 217 | **223** - 217 | **223** - 223 | **223** - 219 | **223** - 219 | **223** - 223 | **223** - 223 | **223** - 217 | **223** - 217 | **223** - 217 | **223** - 221 | **223** - 219 |
| D3S2417 | **261** - 255 | **261** - 255 | **261** - 263 | **261** - 255 | **261** - 255 | **261** - 261 | **261** - 255 | **261** - 255 | **260** - 256 | **260** - 256 | **260** - 260 | **260** - 256 |

*^1^*The size of the conserved disease haplotype is 3.9 Mb.
